# The opinions of adults about the ban on cigarette sales to minors

**DOI:** 10.1186/s12971-016-0104-9

**Published:** 2016-12-05

**Authors:** H. Ozcebe, N. Bilir, E. Inal, H. Unlu, E. Beser, G. Can, E. D. Evci Kiraz, P. Okyay, D. Arslantas, F. Abacigil, V. Senol, E. Turhan, S. Gokgoz, E. O. Calıkoglu, Z. Kocan

**Affiliations:** 1 Institute of Public Health, University of Hacettepe, Ankara, Turkey; 2 Yalova Vocational High School, University of Yalova, Yalova, Turkey; 3 Department of Public Health, Faculty of Medicine, University of Adnan Menderes, Aydın, Turkey; 4 Department of Public Health, Faculty of Medicine, University of Karadeniz Teknik, Trabzon, Turkey; 5 Department of Public Health, Faculty of Medicine, University of Osman Gazi, Eskişehir, Turkey; 6 Department of Public Health, Faculty of Medicine, University of Erciyes, Kayseri, Turkey; 7 Provincial Directorate of Public Health, İzmir, Turkey; 8 Department of Public Health, Faculty of Medicine, University of Kirklareli, Kırklareli, Turkey; 9 Department of Public Health, Faculty of Medicine, University of Ataturk, Erzurum, Turkey

**Keywords:** Children, Tobacco, Tobacco point of sale, Turkey

## Abstract

**Background:**

Selling of tobacco products to minors has been banned since 1996 by the tobacco control law in Turkey. However, it is also important for the public to support practices that prevent the access of tobacco products to minors. In addition, every individual has the responsibility of carrying out society based programs that restrict access to tobacco products especially to children and the youths. Social sensitivity is considered an important factor in the prevention of tobacco use. This study aims to learn about the opinions and attitudes of adults with regards to minors access to tobacco products.

**Methods:**

The study was a descriptive study conducted in nine city centers in Turkey. The total number of participants reached was 3241. The questionnaire was developed by the research team and consisted of 22 questions concerning knowledge and behaviors of adults on restriction of tobacco sales to minors and their observations with regards tobacco sales to minors. Data was collected through face to face interview. Pearson chi-square test was used for the bivariate analysis whereas logistic regression was investigate the relationship between “the participant’s response against tobacco sales to minors” and the following explanatory variables; “age”, “educational status”, “income level”, “working status”, “minors access to cigarettes”, “smoking ratio in high school” and “sales of tobacco to minors”.

**Results:**

More than half of the participants (60.5%) belonged to the age group 25–44 years, 61.3% graduated from high school or university. Most of the participants were smoker (39.2%) or ex-smoker (19.1%), and 41.7% of the participants was non-smoker. A greater proportion of the participants (76.2%) believed that smoking prevalence was greater than 40% among high school students. One in four (27.8%) adults did not know that tobacco control law bans sell of tobacco products to minors in Turkey. More than half of the participants (57.1%) ever witnessed tobacco sales to minors and 63.6% of them did not act when confronted with the event. Almost all (96.8%) of the respondents thought that access of minors to tobacco products was not difficult. The results of logistic regression of participant’s response against tobacco sales to minor and related factors for current smokers showed that respondents who believed smoking ratio in high school was 4–5 adolescent out of 10 (aOR: 1.59; 95% CI: 1.09–2.34) were more likely to give a warning or informing the police or other people as compared to respondents whose perception on the smoking ratio among high school students was 6–7 adolescents out of 10. The results of logistic regression of non-smokers’ response against tobacco sales to minor were who are from higher educational level, higher economic status, working status and who believed smoking ratio in high school was 4–5 adolescent out of 10 and 2–3 adolescent out of 10 were more likely to give a warning or informing the police or other people as compared to the others.

**Conclusions:**

Although laws prohibiting the sale of tobacco products to the under age group is very important with regards to accessibility of minors to tobacco products, most of the study participants believed that minors can still easily access tobacco products, and more than half of the participants did not act when confronted with the event. The education, information and monitoring program most especially as it concerns salesman, should be reviewed and strengthened to obey the rules on sales of tobacco products to minors. Education program should be carried out to increase the knowledge and awareness of the community for sale of tobacco to minors. Social sensitivity is important for the prevention of tobacco use and every individual have a responsibility in carrying out this society based program, most especially as it related to prevention of tobacco usage among children and youths.

## Background

Tobacco use is one of the most important public health concerns worldwide. The rapid increase in health problems associated with tobacco usage have drawn a lot of attention, and great efforts have been put in place to reduce tobacco use [1].

Tobacco control activities first started in developed countries, and later found their place in all countries’ agenda with the World Health Organization (WHO) Framework Convention on Tobacco Control (FCTC) which was accepted at the World Health Assembly in 2003 [2]. FCTC points out two main groups of approaches to reduce tobacco use; the first approach is to reduce the demand for tobacco products, whereas, the second one is to reduce the supply of tobacco products, including placing ban on the sale of tobacco products to children and youth [3, 4]. According to several studies, increased enforcement of laws that ban tobacco sales to minors can reduce access of minors to tobacco products [5–7]. In an observational study on the impact of anti-smoking legislation in Woodridge, a suburban community of Chicago, it was found that both merchant sales and adolescent smoking behavior were reduced after the passage of the law [6]. According to the study, the rates of cigarette experimentation and regular use of cigarettes by adolescents were reduced by over 50% between the pre- and post-test observation periods. In addition, the reduction in cigarettes use was still apparent after 7 years [6]. Though previous studies suggest that the enforcement of laws banning the sale of tobacco products to the underage can lead to a reduction in tobacco use among minors, by making it harder to purchase cigarettes [7]. However, the effectiveness of tobacco control laws is undermined by other “social sources” of cigarettes [8]. For example, an intervention study done by Forster and colleagues to enact local ordinances, change retail merchants’ behavior, and promote enforcement of laws prohibiting illegal sales to minors in 14 Minnesota communities found that the prevalence of smoking among the young age groups was much less in the intervention communities as compared to other communities. In addition, they also observed that youths who reported ever smoking were very likely to cite social sources for cigarettes, though, older youth and those reporting weekly smoking also reported purchasing their own tobacco [5]. There are also similar studies showing that tobacco laws alone are not enough to reduce illegal sales to minors [8–10].

Public support for practices on the prevention of access to tobacco products for minors is very important if any progress is to be made. Studies have been done to this effect [11–13]. A 1992 national telephone poll of 1200 adults residing in New York showed that approximately 83% were in favor of legislations banning tobacco advertisements targeted at teenagers in New York. In addition, three quarters of smokers also supported a ban on tobacco advertisements targeted at teenagers [14]. Another study shows that most of the American public do not believe that laws prohibiting the sale of tobacco to minors are adequately enforced [15]. In a survey conducted in the United States, it was reported that 8 out of 10 adults felt it was “very easy” or “somewhat easy” for teenagers to buy cigarettes near where they live [16]. Some literatures suggest that majority of adults think laws banning the sale of tobacco products to minors have not been adequately enforced [15, 17, 18].

Tobacco control activities first started in Turkey through the Law on Prevention of Harms of Tobacco Use in 1996 [19]. This Law was further amended in 2008, and played a role in making Turkey the third country in the world to be 100% smoke-free country [20]. The first and the amended law banned selling of tobacco products to minors less than 18 years of age [19, 21]. Even though there are some difficulties in the implementation of this item, Turkey has recorded important achievements in tobacco control and is the first and single country in the world implementing all six strategies of MPOWER with success [22]. However, tobacco use is still considered to be one of the main public health issues in Turkey, as Turkey is one of the countries with the highest tobacco usage rate in the world [22].

The frequency of smoking is 27.1% among older than 15 years in Turkey and about 15 million people use cigarette and other tobacco products [22]. According to the Global Adult Tobacco Survey (GATS), one fifth of smoker adults start using tobacco products before 15 years and more than half of adults start using tobacco products before 18 years [22]. This result reveals that tobacco use is quite prevalent among the young, also, a great number of smokers starts this behavior during their youth. Thus, it is very important to prevent children and the young from starting tobacco use.

The Turkish Law on the Prevention and Control of Hazards of Tobacco Products, Law No. 4207 has an article about the “prohibition of access to tobacco use for individuals who are under the age of 18 through sale and distribution” [19]. This is a very important law with regards to tobacco control as during tobacco control among minors, one of the focus is to prevent minors from accessing tobacco products with emphasis on advertising bans, sponsorship, promotion, brand stretching and regulations aimed at public awareness and point of sales [19]. This study aims to learn about the opinions and attitudes of adults with regards to minors access situations to tobacco products.

## Methods

### Study design and data collection

The study was a descriptive study conducted in nine city centers of Turkey; Ankara, Aydın, Erzurum, Eskisehir, İzmir, Gümüşhane, Kayseri, Kırklareli, and Trabzon. These cities were selected by convenience from different geographic regions of the country. Thus, the study findings are not representative of the whole country.

The study was organized by the Institute of Public Health, University of Hacettepe. Faculty members from the departments of public health or public health specialist in each city coordinated field data collection. The study was funded by Hacettepe University Scientific Research Projects Coordination Unit.

The sample size was determined by accepting the prevalence as 0.50 and error as 0.05 (with 95% confidence interval). Participation in the study was voluntary and convenience sampling technique was used with the aim of reaching 400 people in each city center. Participant selection criteria included adults aged 18 years and above.

The questionnaire form was developed by the study team, based on previous literatures. The questionnaire consisting of 22 questions regarding smoking behavior of the participants, knowledge of tobacco control law, observation and reactions on tobacco sales to minors. The items in the questionnaire consisted of socio-demographic characteristics of participants (6 questions), questions on individual smoking profile (4 questions), participants opinion about smoking among minors (5 questions), knowledge about prohibition of sales of tobacco to minors (2 questions), ever witnessed tobacco sale to minors and their reactions (3 questions) and suggestion about tobacco sale to minors (2 questions). The questionnaire was initially pretested and data was collected through face to face interview.

The questionnaire was initially pretested and data was collected through face to face interview. All interviewers were trained by the local research team in each city. The interviewers comprised university graduates or final year students. The expected total number of people to be interviewed was 3600 however, the total number of people reached was 3241.

- Variables (i) *Socio-demographic variables;* Socio demographic variables included age, educational status, working status and income level. Age was recorded into 18–24, 25–34, 35–44, 45–54 and 55 and above years. Educational status was recorded into literate, primary (5 years), secondary (8 years), high school (11 years), university and others. Working status was grouped into working and not working. Income level was grouped into 5 groups: income level was grouped into lowest, lower middle, middle, higher middle and highest.
- (ii) *Perception and knowledge of tobacco use and sales to minor and tobacco control law covariates;* this included questions on smoking ratio in high school, sales of tobacco to minors, penalty for sales of tobacco to minors, the places providing cigarettes to minors, and minors access to cigarettes. Smoking ratio in high school was recorded into the following groups; one adolescent out of 10, 2–3 adolescents out of 10, 4–5 adolescents out of 10, and 6–7 adolescents out of 10. Sales of tobacco to minors were grouped into prohibited, allowed with their parents and not prohibited. Penalty for sales of tobacco to minors was categorized into fine, cancellation of sales certification, and imprisonment, the places providing cigarettes to minors were grouped into grocery/market, friends, vendor, and family. Minors’ access to cigarettes was grouped into very easy, not very difficult and very difficult.
- (iii) *Ever witnessed tobacco sales to minor and response against the situation variables;* Respondents were asked if they witnessed any tobacco sale to minors, the sellers response to minor and the participants response against tobacco sales to minor. Ever witnessed any tobacco sales to minors was grouped into yes and no. The sellers response to minor was categorized into asking his/her ID card and not asking anything and selling tobacco product. The participant’s response against this event include the following categories; Doing nothing, warning the sellers, warning the owner, warning the child, informing the police, and talking to the other people about this event.
- (iv) *Smoking status:* Smoking status was grouped into current smokers, former smokers and never smoked.

### Statistical analysis

For the study sample characteristics, variables were reported as frequencies and percentages. Pearson chi-square tests were conducted to examine bivariate differences with smoking status where the assumption of the expected count less than 5 should not exceed 20% for variables was satisfied, if otherwise exact chi-square test was used. “Participant’s response against tobacco sales to minor” was specified as dependent variable and dichotomized into warming/informing and nothing (as reference). Additionally, independent variables which includes “age”, “educational status”, “income level”, “minors access to cigarettes” and “sales of tobacco to minors” were grouped as follows: “Age” was classified into 18–24, 25–34 and 35 and above; “educational status” was classified into secondary and below, high school and university; “income level” was classified into middle and below, and higher middle and above; “minors access to cigarettes” was classified into very easy, not very difficult and very difficult and “sales of tobacco to minors” was classified into prohibited and not prohibited, allowed with their parents. Since “smoking status” was thought the most important factor affecting the behavors, two logistic regression models were performed to identify the relationship between “participant’s response against tobacco sales to minor” and related factors for current smokers and participants who never smoked. For current smokers, logistic regression model was constructed to identify the relationship between “participant’s response against tobacco sales to minor” and the following explanatory variables; socio-demographic variables (age, educational status, income level, working status), “minors access to cigarettes” and “smoking ratio in high school”. For participants who never smoked, logistic regression model was constructed to identify the relationship between “participant’s response against tobacco sales to minor” and the following explanatory variables; socio-demographic variables (age, educational status, income level, working status), “smoking ratio in high school” and “sales of tobacco to minors”. Reference categories were shown in Tables 4 and 5. Hosmer-Lemeshow statistics was used to assess model assumption. The variables which were found as significant according to Pearson chi-square test were included into logistic regression model as independent variables. Since some participants did not answered all the questions, there were some missing observations in the dataset. While performing multiple logistic regression, list wise deletion method was used to handle with missing observations. A *p*-value below 0.05 was accepted as significant.

### Ethics

This study was approved by Non-interventional Clinical Researches Ethics Board of Hacettepe University (GO 13/242-19).

## Results

Most of the participants were smoker (39.2%) or ex-smoker (19.1%), and 41.7% of the participants was non-smoker; 16.1% of the smokers started smoking daily before the age of 15 while 42.6% started smoking between ages 15–17 (data is not given in the table).

A greater proportion of respondents (60.5%) were in the age group 25–44 years age group, approximately 61.3% of participants were high school or university graduates. A greater percentage of study participants (65.9%) were currently working. About 85.7% had middle and below income level. More than half of the participants (55.5%) who were current smokers were in the age group 18–34 years. Participants who were never smoked had mostly high school and above educational status (64.0%), whereas more than half of the participants (64.7%) who were current smokers had high school and above educational status. Most of the participants who were never smoked, smoked and quit, current smokers had middle income level (43.7, 49.0 and 43.7% respectively) (Table 1).

**Table 1:** Some sociodemographic characteristics of participants

Socio-demographic characteristics	Smoking Status						Total		*P*
	Never		Smoked and quit		Current smoker		N	%	
Age	n	%	n	%	n	%			
18–24	275	22.3	49	8.7	187	16.0	511	17.2	<0.001
25–34	483	39.2	171	30.3	462	39.5	1116	37.6	
35–44	267	21.7	113	20.0	299	25.5	679	22.9	
45–54	138	11.2	123	21.8	145	12.4	406	13.7	
55 and over	68	5.5	109	19.3	78	6.7	255	8.6	
Total	1231		565		1171		2967	100.0	
Educational Status									
Literate	123	9.6	45	7.8	71	5.9	239	7.8	<0.001
Primary (5 years)	122	9.6	97	16.7	164	13.7	383	12.5	
Secondary (8 years)	214	16.8	112	19.3	234	19.5	560	18.3	
High school (11 years)	345	27.0	160	27.6	389	32.5	894	29.3	
University	453	35.5	165	28.4	326	27.2	944	30.9	
Others (vocational school, etc.)	19	1.5	1	0.2	13	1.1	33	1.1	
Total	1276		580		1197		3053	100.0	
Working Status									
Working	768	61.0	375	65.4	847	71.7	1990	66.0	<0.001
Not working	492	39.0	198	34.6	335	28.3	1025	34.0	
Total	1260		573		1182		3015	100.0	
Income Level									
Lowest	72	5.7	36	6.1	60	5.0	168	5.5	<0.001
Lower middle	486	38.4	199	34.0	400	33.5	1085	35.6	
Middle	553	43.7	287	49.0	522	43.7	1362	44.7	
Higher middle	129	10.2	52	8.9	169	14.2	350	11.5	
Highest	25	2.0	12	2.0	43	3.6	80	2.6	
Total	1265		586		1194		3045	100.0	

Note: Some participants did not answer all the questions

All the participants believed that smoking behavior was highly prevalent among adolescents. Only 7.2% of participants believed that one out of 10 high school students were smokers, most of the participants (76.2%) believed that smoking prevalence was more than 40% among high school students. Participants who were current smokers thought that more high school students were current smoking as compared to non-smokers (*p* = 0.001) (Table 2).

**Table 2:** The perception and knowledge of tobacco use and sales to minor and tobacco control law by smoking status

Perception and Knowledge on Tobacco Use and Sales	Smoking Status						Total		*P*
	Never		Smoked and quit		Current smoker		N	%	
The smoking ratio in high school	n	%	n	%	n	%			
One adolescent out of 10	88	7.1	44	7.8	80	7.0	212	7.2	0.001
2–3 adolescents out of 10	240	19.3	90	15.9	161	14.0	491	16.6	
4–5 adolescents out of 10	456	36.7	196	34.7	385	33.5	1037	35.0	
6–7 adolescents out of 10	460	37.0	235	41.6	523	45.5	1218	41.2	
Total	1244		565		1149		2958	100.0	
Sales of tobacco to minors									
Prohibited	888	72.5	408	72.3	837	71.8	2133	72.2	0.700
Allowed with their parents	120	9.8	61	10.8	107	9.2	288	9.8	
Not prohibited	216	17.6	95	16.8	221	19.0	532	18.0	
Total	1224		564		1165		2953	100.0	
Penalty for sales of tobacco to minors									
Fine	565	58.7	240	53.9	508	55.3	1313	56.4	0.003
Cancellation of sales certification	256	26.6	140	31.5	310	33.8	706	30.4	
Imprisonment	142	14.7	65	14.6	100	10.9	307	13.2	
Total	963		445		918		2326	100.0	
Major point were cigarettes are provided to minors									
Grocery/Market	554	44.7	265	47.4	456	40.0	1275	43.4	<0.001
Friends	524	42.3	217	38.8	454	39.8	1195	40.7	
Vendor	108	8.7	52	9.3	169	14.8	329	11.2	
Family	52	4.2	25	4.5	62	5.4	139	4.7	
Total	1238		559		1141		2938	100.0	
Minors access to cigarettes									
Very easy	749	59.3	350	60.3	795	66.9	1894	62.5	0.001
Not very difficult	477	37.8	209	36.0	354	29.8	1040	34.3	
Very difficult	37	2.9	21	3.6	40	3.4	98	3.2	
Total	1263		580		1189		3032	100.0	

Note. Some participants did not answer all the questions

A higher percentage of the participants believed that it was prohibited to sell tobacco to minors, however, 9.8% of respondents believed that minors were allowed to buy tobacco products with their parents and 18.0% thought that there were no laws prohibiting the sale of tobacco to minors. A greater proportion of respondents believed that the penalty for sales of tobacco to minors was fine (56.4%) and a greater percentage of current smokers (55.3%) as compared to non-smokers believed so (*p* = 0.003) (Table 2).

Almost half of the participants (54.6%) believed that minors accessed tobacco products from grocery/market or vendors, a greater proportion of current smokers as compared to non-smokers believed that minors accessed cigarettes from vendors (14.8 and 8.7%) (Table 2).

Only 3.2% of the participants believed that minors’ access to cigarettes was very difficult. According to smoking status, smokers (66.9%) believed that minors access to cigarettes was very easy as compared to nonsmokers (59.3%) (*p* = 0.001). (Table 2).

More than half of the participants (57.1%) ever witnessed any tobacco sales to minors, current smokers reported higher percentage (63.8%) of having witnessed tobacco sales to minor as compared to nonsmokers (*p* < 0.001) (Table 3).

**Table 3:** Ever witnessed tobacco sales to minor and response against the situation by smoking status

Experience of participants on tobacco sales	Smoking Behavior						Total		*P*
	Never		Smoked and quit		Current smoker		N	%	
	n	%	n	%	n	%			
Ever witnessed any tobacco sales to minors									
Yes	630	50.0	343	59.0	758	63.8	1731	57.1	<0.001
No	631	50.0	238	41.0	430	36.2	1299	42.9	
Total	1261		581		1188		3030	100.0	
The seller’s response to the child									
Asking his/her identity card	76	12.0	51	14.8	80	10.6	207	12.0	0.176
Not asking anything and selling tobacco product	557	88.0	294	85.2	677	89.4	1510	88.0	
Total^a^	633		345		749		1727	100	
The participant’s response against tobacco sales to minors									
Doing nothing	372	60.4	199	59.1	504	68.3	1075	63.6	0.002
Warning the seller’s	98	15.9	61	18.1	84	11.4	243	14.4	
Warning the owner	33	5.4	14	4.2	22	3.0	69	4.1	
Warning the child	77	12.5	46	13.6	89	12.1	212	12.5	
Informing the police	21	3.4	3	0.9	14	1.9	38	2.2	
Talking to the other people about this event	15	2.4	14	4.2	25	3.4	54	3.2	
Total^a^	616		337		738		1691	100.0	

Note: ^a^The participants were witness at any tobacco sale under 18 years old

Approximately 64% of respondents who ever witnessed any tobacco sales to minors did not act when confronted with the event and current smokers made up a higher proportion of the group “doing nothing” as compared to non-smokers, however, nonsmokers had more positive response with regards to warning/informing the police or other people (*p* = 0.002). Approximately 88.0% of the salesmen sold tobacco products without asking the child’s age or asking for an identity card (Table 3).

Two logistic regression models with multiple predictors were constructed to investigate relationship between participant’s response against tobacco sales to minors and related factors for current smokers and participants who never smoked. The results of logistic regression models for current smokers were given in Table 4. The results of logistic regression model showed that the model assumptions were satisfied (*p* = 0.271) and the model was found to be statistically significant (*p* < 0.001). Respondents who believed smoking ratio in high school was 4–5 adolescent out of 10 (aOR: 1.59; 95% CI: 1.09–2.34), were more likely to give a warning or informing the police or other people as compared to respondents whose perception on the smoking ratio among high school students was 6–7 adolescents out of 10, whereas 2–3 adolescent out of 10 (aOR: 0.74; 95% CI: 0.40–1.37) were less likely to give a warning or informing the police or other people (Table 4).

**Table 4:** Logistic regression results of participant’s response against tobacco sales to minor and related factors for current smokers

	OR	*P*	95% CI
Constant	0.15	<0.001	
Educational Status			
Literate/Primary/Secondary (ref)	1		
High School	1.52	0.054	0.99–2.33
University	1.47	0.095	0.94–2.30
Age group			
18–24 (ref)	1		
25–34	0.97	0.914	0.55–1.69
35 and above years	1.70	0.056	0.98–2.95
Income level			
Middle, lower middle,/ lowest (ref)	1		
Highest /higher middle	1.38	0.078	0.96–1.96
Working Status			
Not working (ref)	1		
Working	1.28	0.266	0.83–1.97
Minors access to cigarettes			
Very easy/Not very difficult (ref)	1		
Very difficult	3.11	0.076	0.89–10.89
Smoking ratio in high school			
6–7 adolescents out of 10 (ref)	1		
4–5 adolescents out of 10	1.59	0.016	1.09–2.34
2–3 adolescents out of 10	0.74	0.344	0.40–1.37
One adolescent out of 10	1.94	0.139	0.81–4.67

Classification Rate:71.7%; Hosmer-Lemeshow Test: *p* = 0.271

The results of logistic regression models for participants who never smoked were given in Table 5. The results of logistic regression model showed that the model assumptions were satisfied (*p* = 0.215) and the model was found to be statistically significant (*p* < 0.001). Respondents who believed smoking ratio in high school was 4–5 adolescent out of 10 (aOR: 1.71; 95% CI: 1.12–2.62), and 2–3 adolescent out of 10 (aOR: 2.25; 95% CI: 1.33–3.83) were more likely to give a warning or informing the police or other people as compared to respondents whose perception on the smoking ratio among high school students was 6–7 adolescents out of 10. Having knowledge of the prohibition of tobacco sales to minors (aOR: 2.06; 95% CI: 1.34–3.16) was associated with higher likelihood of giving a warning or informing the police or other people as compared to not having any knowledge on the prohibition of tobacco sales to minors. Participants who had highest or higher middle income level (aOR: 1.86; 95% CI: 1.28–2.72) were more likely to give a warning or informing the police or other people as compared to respondents who had middle, lower middle or lowest income level. Respondents knowing sales of tobacco products prohibited to minors (aOR: 2,06; 95% CI: 1.34–3.16) were more likely to give a warming or informing the police or other people as compared to respondents who were not working (Table 5).

**Table 5:** Logistic regression results of participant’s response against tobacco sales to minor and related factors for participants who never smoked

	OR	*P*	95% CI
Constant	0.16	<0.001	
Educational Status			
Literate/Primary/Secondary (ref)	1		
High School	0.91	0.709	0.57–1.47
University	0.99	0.984	0.62–1.59
Age			
18–24 (ref)	1		
25–34	0.95	0.843	0.56–1.60
35 and above years	0.99	0.988	0.58–1.70
Income level			
Middle, lower middle,/ lowest (ref)	1		
Highest /higher middle	1.86	0.001	1.28–2.72
Working Status			
Not working (ref)	1		
Working	1.23	0.326	0.81–1.88
Sales of tobacco to minors			
Not prohibited (ref)	1		
Prohibited	2.06	0.001	1.34–3.16
Smoking ratio in high school			
6–7 adolescents out of 10 (ref)	1		
4–5 adolescents out of 10	1.71	0.014	1.12–2.62
2–3 adolescents out of 10	2.25	0.003	1.33–3.83
One adolescent out of 10	2.06	0.105	0.86–4.94

Classification Rate: % 64.5; Hosmer-Lemeshow Test: *p* = 0.215

## Discussion

Turkey is known as a country with a history of important successes in tobacco control [23]. The prevention of tobacco access is one the most important intervention, thus, preventing children and youth from starting tobacco. The most important stage in the prevention of accessibility is determined as increasing taxes and applications for point of sales [1, 2].

In this study, 3241 people were interviewed in 9 city centers. The people interviewed are mainly young adults and their educational status was higher than average level of education when compared to the general population. Most of the smoker and ex-smoker participants declared that they started daily smoking at adolescent ages. The participants in the current study are also aware of this as they believed that smoking is a very common behavior among high school students. According to GATS (2012), among ever daily smokers who were aged 18–34 years, 17.1 years was the average age daily smokers started smoking [22]. These findings actually support that tobacco use is accepted as a public health problem among young people.

As earlier stated, one of the most important intervention for the prevention of tobacco use among youths is the prevention of accessibility. In consonance, previous studies have shown that enforcement of laws against tobacco sales to minors is an effective strategy to maintain a barrier for accessibility of tobacco in the community [5, 6, 8]. WHO FCTC also has some suggestion on the enforcement of tobacco sales to minor through regulations of point of sales [24]. The regulation on tobacco sale to minors in Turkey is old, it had been prohibited since 1996, prison sentence was later added as a new regulation in 2008 [19, 21]. In this study, 72.2% of the all respondents gave the true answer with regards rule on tobacco sales to minors, there is not any difference of their knowledge on rule of sales of tobacco to minors between smokers and non-smokers. However, the result of our findings shows that prison sentence is less known (13.2%) and goes to support the fact that information about prison sentence being a part of the law has not become widespread in the community, especially among the smokers. The way of giving information about tobacco selling ban to minors is by having poster at points of sales as well as inspection of this sale points to see if they have the ban posters in the country. However, the posters have only information showing that tobacco sell is banned to children less than 18 years of age. The results of this study revealed that this intervention has been quite effective in increasing the awareness of adults about tobacco selling ban to minors, but this intervention was not effective in increasing the knowledge on pertaining prison sentence. It is worth noting that a high-level debate has been ongoing among the leaders of tobacco control in the country. Some are of the opinion that informative program about the ban of cigarette sale to children under the age of 18 can create a wrong perception as this information can encourage minors to be eager to smoke after the age of 18. Because of this high-level debate, there is not any proactive campaign carried out on sale of tobacco to youth, this is a barrier in front of the distribution of the true information widely in the country.

Literatures show that enforcement of tobacco sales ban to children decreases accessibility of minors to tobacco products [5, 6, 8]. In this study, 66.7% of the smokers and 59.3% of non-smokers think that accessibility of tobacco products is very easy and a larger proportion of respondents assume that individuals who are under the age of 18 can buy tobacco from markets or vendors. Vendors are mentioned by smokers more than non-smokers and ex-smokers (*p* < 0,001). GYTS in Turkey also supports the results of this study, it states that 53.7% of the youth buy cigarettes from markets and 79.1% of young smokers are not refused sell [25]. GYTS results are in consonance with our study finding. There are also other supporting studies [15, 26, 27]. According to Elders, laws prohibiting the sale of tobacco to minors are not adequately enforced [15]. Also, Marcus and colleagues in their study found that 8 out of 10 adults believed teenagers can easily reach tobacco products “very easily” or “somewhat easily” in their neighborhood [16]. Enforcement of laws against tobacco sales to minors should be monitored and strengthened at groceries and vendors [28]. Changing retail merchants’ behavior to prevent illegal sales of tobacco to minors should be enforced and promoted [5]. In Turkey, even though the salesman are informed about the regulation on the sales of tobacco yearly, still children are able to access tobacco products from markets and vendors. As such, in the country, the education, information and monitoring program most especially as it concerns salesman and owners of vendors, should be reviewed and strengthened to obey the rules on sales of tobacco products to minors.

Previous studies have shown that youth can use social sources to access tobacco product [5, 8–11]. Our study findings are in consonance, the result of our study shows that adults are also aware of children accessibility of tobacco products through families and friends.

Public support in the enforcement of laws governing tobacco sales is also important if preventing the access of tobacco product to minors is to be achieved. In our study, 63.8% of the smokers, 59.0% of ex-smokers and 50.0% of non-smokers declared that they witnessed cigarette sale to minors, among all witness participants, 88.0% said the sellers’ response was not asking anything and selling the tobacco product. Also, most of the participants did not take any action, the percentage of smokers not taking any reaction was much higher than non-smokers. More ex-smokers reacting this event is than smokers and non-smokers, they would have had some health problems because of smoking in the past. In a society where there is a target of preventing the sale of cigarette to minors, the salesman have a role to play, in addition, it is expected that all other individuals in the society should be sensitive and play a role in the monitoring of sale points of tobacco. Increasing the awareness of society in terms of age restrictions with regards tobacco sales will strengthen the practicality of tobacco laws. There are ongoing debate in the USA about the drawing of age limit to 21 for tobacco sale, however, it is suggested that increasing the awareness of society and in overall, decreasing the usage of tobacco should be of paramount importance [12, 29]. Social sensitivity is important for the prevention of tobacco use and every individual have a responsibility in carrying out this society based program, most especially as it related to prevention of tobacco usage among children and youths.

A higher proportion of smoker respondent who believed the smoking ratio in high school was 6–7 adolescent out of 10, believed that major point were cigarettes are provided to minors were vendor and family, believed that minor access to cigarette was easy, ever witnessed tobacco sale and doing nothing against tobacco sales to minor belonged to the current smokers categories, Also, a lower proportion of respondent who believed imprisonment was the penalty for sale of tobacco to minors belonged to the current smokers category. This finding goes to show that current smokers as compared to other categories believed that tobacco use among minor is prevalent and access is easy, however as compared to other smoking categories were less aware that prison sentence was a part of the law, they also were more aware that minors can access tobacco product through social source such as the family, in addition, current smokers witnessed more sales of tobacco product to minors and did nothing. Non-smokers and highly informed people with regards to tobacco control law intervened more when they witness tobacco sales to minors. In the prevention of smoking among minors, adults should not only be role models for children but they should also warn them about the right behaviors directly. Because of this behavioral difference, two logistic model were conducted.

The results of logistic regression of participant’s response against tobacco sales to minor and related factors for current smokers showed that sociodemographic characteristics of participants did not affect the behavior of the person. Only, respondents who believed smoking ratio in high school was 4–5 adolescent out of 10 (aOR: 1.59; 95% CI: 1.09–2.34) were more likely to give a warning or informing the police or other people as compared to respondents whose perception on the smoking ratio among high school students was 6–7 adolescents out of 10. According to this results of model, we could not explain the witness smokers’ reaction to sale of cigarettes with sociodemographic factors and their knowledge on tobacco sale items of the law. It is need the psychosocial behavior theories should be studied on smokers behaviors to understand the causes under their behaviors to strengthen the response of smokers. On the other hand, smoking cessation interventions would change the behaviors of smokers when they witness tobacco sales to minors.

The results of logistic regression of non-smokers’ response against tobacco sales to minor were who are from higher educational level, higher economic status, working status and who believed smoking ratio in high school was 4–5 adolescent out of 10 and 2–3 adolescent out of 10 were more likely to give a warning or informing the police or other people as compared to the others. The people from higher socioeconomic status could take more responsibility to monitor the sale of tobacco products to minors. Information and education interventions on tobacco sale to minors would be given to the community, especially to low socioeconomic status in the community. Additional interventions aimed at individual responsibilities are needed for individual smokers about the ban of cigarette sale to children as earlier stated.

### Study limitations

This study has some limitations. Firstly, the study finding is not representative for the whole country. Sampling of cities and the people interviewed were through convenient sampling, as such, the results should be carefully interpreted. Secondly, the average duration of questionnaire completion was approximately 3–4 min, perhaps, due to the time duration some questions were left unanswered by the participants. During data entry, some of these questions were removed and not used in this study.

## Conclusions

Though adults think that tobacco use is a prevalent among minors, tobacco product sales ban is an important subject in tobacco accessibility, most especially at points of sales, unfortunately not enough is known by the adults and they do not take enough responsibility in ensuring the implementation of the tobacco control law as it pertain access to tobacco products by minors. Though Turkey has already made important accomplishments with regards to tobacco control, there is still a need for detailed studies and some interventions are needed to reduce accessibility of minors to tobacco products.

## Abbreviations

FCTC: Framework Convention on Tobacco Control
GATS: Global Adult Tobacco Survey
GYTS: Global youth tobacco survey
WHO: World Health Organization
